# P01-005 – Idiopathic uveitis and FMF

**DOI:** 10.1186/1546-0096-11-S1-A9

**Published:** 2013-11-08

**Authors:** F Salehzadeh, O Yasrebi, S Jahangiri, S Hosseiniasl

**Affiliations:** 1 Pediatric Rheumatology, Bouali Hospital, Iran, Islamic Republic Of; 2 Pediatric Rheumatology, Arums, Iran, Islamic Republic Of; 3 Genetic, Emam Khomeini hospital, Ardabil, Iran, Islamic Republic Of

## Introduction

Familial Mediterranean fever (FMF) is an auto inflammatory disease characterized by attacks of fever and polyserositis. FMF is often associated with other autoimmune diseases such as rheumatoid arthritis, PAN, Behcet.

## Objectives

Uveitis is an inflammatory process of eyes caused by underlying infectious and inflammatory disorders. This study investigates the probable relationship between Idiopathic Uveitis and familial Mediterranean fever.

## Methods

Patients with idiopathic uveitis that didn't have Infectious and inflammatory causes referred to the genetic laboratory to12 common MEFV genes analysis. P369S, F479L, M680I(G/C), M680I(G/A), I692del, M694V, M694I, K695R, V726A, A744S, R761H, E148Q mutations were analysed by using amplification refractory system for 11 of those and the PCR was performed for E148Q.

## Results

12 patients with idiopathic uveitis were enrolled in this study. 10 of them were female and 2 were male. The youngest patient was a 7-year-old child and the oldest was 57. The most common complaints of patients was blurred vision and then eye redness. One patient was heterozygous for Wt/R761H in the MEFV genetic analyses. Genetic analysis of 12 most common MEFV mutations in the patients with idiopathic uveitis didn't have any positive results.

## Conclusion

According to the analysis of 12 most common MEFV gene mutations, FMF is not an underlying cause of idiopathic uveitis. On the other hand uveitis merely could not be the first presentation of FMF.

## Disclosure of interest

None declared

